# Plasma soluble tumor necrosis factor receptor I as a biomarker of lupus nephritis and disease activity in systemic lupus erythematosus patients

**DOI:** 10.1080/0886022X.2023.2174355

**Published:** 2023-03-22

**Authors:** Xin-ran Liu, Yuan-yuan Qi, Ya-fei Zhao, Yan Cui, Zhan-zheng Zhao

**Affiliations:** a Nephrology Hospital, the First Affiliated Hospital of Zhengzhou University, Henan, China; b Institute of Nephrology, Zhengzhou University, Henan, China

**Keywords:** Systemic lupus erythematosus, lupus nephritis, tumor necrosis factor receptor, disease activity

## Abstract

**Objectives:**

The goal of our study was to evaluate the potential role of sTNF-RI as a biomarker of renal involvement in SLE patients and active SLE.

**Methods:**

The study sample consisted of two cohorts. The discovery cohort included 16 SLE patients without renal involvement (non-LN), 60 lupus nephritis (LN) patients and 21 healthy controls (HCs) and the replication cohort included 18 SLE non-LN patients, 116 LN patients and 36 HCs.

**Results:**

The sTNF-RI levels differed significantly in the discovery cohort. The plasma sTNF-RI levels were higher in LN patients than in non-LN patients (*p* = .009) and HCs (*p* = 4 × 10^−6^). Plasma sTNF-RI levels were significantly higher in non-LN patients than in HCs (*p* = .03). The finding was confirmed in independent replication cohort (LNs *vs*. non-LN, *p* = 4.053 × 10^−7^; LNs *vs*. HCs, *p* = 2.395 × 10^−18^; non-LN *vs.* HCs, *p* = 2.51 × 10^−4^). The plasma sTNF-RI levels were associated with disease activity, renal function in SLE patients and urine protein in LN patients. The multivariate analysis revealed that high sTNF-RI was an independent risk factor for renal involvement. The multivariate logistic regression results suggested that high TNF-RI, high systolic blood pressure, high serum creatinine, low C4 and positive anti-dsDNA were independent risks of active SLE patients. A nomogram was constructed based on the results of multivariate logistic regression analysis and it was practical in predicting the risk of the active SLE patients. Immunohistochemistry suggested that the expression of TNF-RI in the kidney was increased.

**Conclusions:**

Plasma sTNF-RI might be a good biomarker of renal involvement and disease activity in SLE patients.

## 1. Introduction

Systemic lupus erythematosus (SLE) is an autoimmune disorder that is characterized by the disruption of immune tolerance and autoantibody formation. It can involve many organs and systems, including the skin, kidney and hematopoietic system [1]. Lupus nephritis (LN) is a common complication and is associated with a poor prognosis in SLE. Asians reportedly have a higher incidence of LN [2], and the early diagnosis of LN is critical for timely management and patient survival. Kidney biopsy is the gold standard for diagnosing LN, but it is invasive [3,4]. Currently, renal involvement in SLE is usually evaluated by urinalysis or by measuring serum creatinine or eGFR. Therefore, finding earlier biomarkers is very important for the timely prediction of renal impairment in SLE patients.

Cytokines, particularly tumor necrosis factor-α (TNF-α), play an essential role in the pathogenesis of SLE. Previous studies had shown that SLE patients had high levels of serum TNF-α, and TNF-α was correlated with disease activity in SLE [5]. Increased renal expression of TNF-α was observed in lupus mice. Tumor necrosis factor receptor I (TNF-RI), the classic ligand for TNF-α, is correlated with chronic diseases, including autoimmune diseases. TNF-RI is ubiquitously expressed on almost all cell types in humans and plays an important role in proinflammatory effects. Moreover, it can activate signaling pathways that lead to cell death [6,7].

Accumulating evidence indicates the involvement of TNF-RI in the development of SLE. Sharapova et al. reported that autoantibodies from SLE patients interact with TNF-RI, thereby inducing murine fibroblast cell death [8]. In a retrospective study of Caucasians, soluble TNF-RI (sTNF-RI) was identified as a potential marker to distinguish SLE patients without renal impairment from LN patients [9].

The goal of our study was to evaluate the potential value of sTNF-RI as a biomarker for the prediction of renal involvement and renal function in Chinese SLE patients.

## 2. Materials and methods

### 2.1. Study population

SLE patients who were hospitalized at the Nephrology Hospital of the First Affiliated Hospital of Zhengzhou University between July 2019 and February 2021 were enrolled. Patients with tumors, pregnancy or other autoimmune diseases were excluded. The study sample consisted of two cohorts. The discovery cohort included 60 LN patients, 16 SLE patients without renal involvement (non-LN) and 21 healthy controls (HCs). The replication cohort included 116 LN patients, 18 non-LN patients and 36 HCs. All of the SLE patients met the American College of Rheumatology (ACR) revised criteria [10] as updated in 1997 [11]. The patients with LN were confirmed by renal biopsy and classified based on the current International Society of Nephrology/Renal Pathologic Society (ISN/RPS) 2003 criteria [12]. All patients were recruited from the First Affiliated Hospital of Zhengzhou University. All subjects were fully informed about the characteristics and purpose of our study and provided informed consent. Our study was approved by the Ethical Committee of the Medical Ethics Committee of Zhengzhou University First Hospital (2019-KY-134).

We collected clinical data at the time of collecting the samples and the data of pathological characteristics at the time of renal biopsy. The disease activity (SLEDAI) was assessed by the SLE Disease Activity Index 2000 [13]. In addition, we also calculated the estimated glomerular filtration rate (eGFR) by using the Modification of Diet in Renal Disease Study equations (eGFR-MDRD) [14] and Chronic Kidney Disease Epidemiology Collaboration equations (eGFR-EPI) [15].

### 2.2. Definition of renal impairment

Renal impairment is defined by the following: 1. Urine protein/creatinine ratio ≥ 0.2; 2. Persistent proteinuria ≥ 0.15 g/d or greater than 1+ by dipstick; 3. Cellular casts including red blood cells (RBCs), hemoglobin, granular, tubular, or mixed, and cellular casts limited to RBC or WBC casts can be substituted for cellular casts in the absence of infection); 4. Active urinary sediment: 5 RBCs/HPF, 5 white blood cells (WBCs)/HPF; and 5. Renal biopsy supports the diagnosis of LN.

### 2.3. Determination of plasma TNF-RI levels

Peripheral blood samples were collected with EDTA-K2 anticoagulant tubes after an overnight fast. Plasma was obtained by centrifugation at 3000 rpm for 10 min within 1 h. All samples were aliquoted and stored at −80 °C until this analysis. The levels of plasma sTNF-RI in the discovery cohort were detected and quantified by Quantibody® Human Inflammation Array 3 (Cat# QAH-INF-G3-4, Raybiotech), and the levels of plasma TNF-RI in the replication cohort were detected and quantified by the Human TNF-RI Quantikine ELISA Kit (R&D Systems, Minneapolis, MN) according to the manufacturer’s instructions.

### 2.4. Immunohistochemistry of TNF-RI expression

Renal lesion tissues were harvested from a renal tissue biopsy, and normal kidney tissue samples adjacent to renal cancer served as normal controls. The deposition of TNF-RI in the kidneys of LN patients with different types was detected by immunohistochemistry (IHC). Two class II LN patients, 1 class III LN patient, 2 class IV LN patients, 3 class V patients and 5 normal controls were included. The renal tissues were prepared into 4 µm thick paraffin sections using polyclonal rabbit anti-human TNR-RI (21574-1-AP, Proteintech) at a 1:100 dilution. The TNF-RI-positive area was quantified using the ImageJ analysis system.

### 2.5. Statistical analysis

χ2 tests or Fisher’s exact tests were used for categorical variables, and data are presented as n, %. Mann–Whitney tests or independent-samples T tests were used for continuous variables, and data are presented as the mean ± standard deviation (SD) or median and interquartile range (IQR). Pearson’s correlation coefficient was applied to detect the correlation between the level of plasma sTNF-RI and clinical indicators. Univariate and multivariate logistic regression analyses were used to explore the variables that were independently related to LN and active SLE. Then, the nomogram was generated based on multivariate logistic regression. In addition, calibration curves, decision curve analysis (DCA) and receiver operating characteristic (ROC) curves were plotted to determine the reliability of our nomogram. All data were analyzed by using R software version 4.1.2, and *p* < .05 was considered significant.

## 3. Result

### 3.1. Study population characteristics

The baseline characteristics of the patients and healthy controls are shown in Table 1. In the discovery cohort, non-LN patients had a median disease duration of 6.5 months (range 1–51.75 months), and the LN patients had a median disease duration of 12 months (range 1–63.25 months). In the replication cohort, the SLE without renal involvement patients had a median disease duration of 6.5 months (range 1–33.25 months), and the LN patients had a median disease duration of 12 months (range 1–48 months).

**Table 1.** Clinical data of SLE patients and healthy controls the discovery cohort and the replication cohort.

Characteristics	The discovery cohort			The replication cohort		
	SLE without renal impairment (*N* = 16)	LN (*N* = 60)	Healthy controls (*N* = 21)	SLE without renal impairment (*N* = 18)	LN (*N* = 116)	Healthy controls (*N* = 36)
Female, n, %	14, 87.5%	50, 83.33%	14, 66.67%	16, 88.89%	92, 79.31%	27, 75%
Age (year), mean ± S.D.	31.81 ± 13.38	32.82 ± 12.42	25.25 ± 3.01	30.67 ± 13.01	34.84 ± 13.68	25.25 ± 3.01
Disease duration (month), median (IQR)	6.5 (1–51.75)	12 (1–63.25)		6.5 (1–33.25)	12 (1–48)	–
Onset age (year), median (IQR)	29 (10–39.25)	29.5 (18.75––37.5)		28 (19.75–37.75)	29 (21–42)	–
SBP (mmHg), median (IQR)	117.5 (106.75–120)	122 (116–135)	114 (106.5–118)	119 (107.25–123)	125 (120–135)	111.5 (105–117)
DBP (mmHg), median (IQR)	76 (69–80.25)	80 (75–85)	78 (71.5–80.5)	75.5 (69–80)	80 (75–86.75)	74.5 (70.5–79.75)
Malar Rash, n, %	6, 37.5%	2, 3.33%	–	8, 44.44%	6, 5.17%	–
Arthritis, n, %	1, 6.25%	3, 5%	–	1, 5.56%	5, 4.31%	–
Serositis, n, %	1, 6.25%	13, 21.67%	–	1, 5.56%	28, 24.14%	–
Fever, n, %	3, 18.75%	1, 1.67%	–	3, 16.67%	3, 2.59%	–
Neurologic disorder, n, %	0, 0%	2, 3.33%	–	0, 0%	1, 0.86%	–
Leukopenia, n, %	4, 25%	3, 5%	–	5, 27.78%	10, 8.62%	–
Lyphopenia, n, %	6, 37.5%	23, 38.33%	–	6, 33.33%	47, 40.52%	–
Thrombocytopenia, n, %	1, 6.25%	5, 8.33%	–	1, 5.56%	14, 12.07%	–
Urea (mmol/l), median (IQR)	3.8 (3.1–5.58)	5.6 (4.03–8.48)	–	4.45 (3.1–5.63)	5.9 (4.43–8.5)	–
Serum creatinine (umol/l), median (IQR)	55.5 (40.75–64.75)	64 (56–90.25)	51 (44–60.5)	54.5 (42.25–64.25)	66.5 (56–99)	51.5(47.25–58.75)
Uric acid (umol/l), median (IQR)	223 (188.25–263.5)	300.5 (258.5–395.25)	–	234 (196.75–258.5)	309.5 (266.75–393.75)	–
eGFR-EPI (ml/min/1.73m2), median (IQR)	124.06 (103.07–144.31)	106.79 (76.77–121.55)	132.11 (126.35–139.68)	126.85 (103.78–140.88)	104.32 (73.17–121.35)	127.67 (122.09–134.13)
eGFR-MDRD (ml/min/1.73m2), median (IQR)	142.92 (107.86–202.86)	111.03 (75.78–131.64)	160.76 (147.19–198.54)	142.92 (111.1–202.68)	102.85 (72.34–133.04)	149.66 (125.35–168.34)
Serum albumin (g/l), median (IQR)	41.6 (39.08–46.45)	36.9 (30.23–40.45)	–	40.5 (37.45–46.35)	34.05 (25.9–38.28)	–
T-CHO (mmol/l), mean (S.D.)	3.74 (3.04–4.91)	3.9 (3.36–5.14)	–	3.74 (3.06–4.7)	4.34 (3.58–5.54)	–
TG (mmol/l), median (IQR)	1.18 (1.03––1.89)	1.31 (0.94–1.94)	–	1.22 (1.05–2.03)	1.47 (1.04–2.09)	–
Decreased C3, n, %	7, 43.75%	12, 20%	–	9, 50%	40, 34.48%	–
Decreased C4, n, %	7, 43.75%	12, 20%	–	9, 50%	30, 25.86%	–
Positive anti-ANAs, n, %	15, 93.75%	54, 90%	–	17, 94.44%	109, 93.97%	–
Anti-dsDNA Ab (U/ml), median (IQR)	37.55 (10.85–135.2)	20.3 (10–169.79)	–	45.45 (12.55–152)	36.89 (10–174.15)	–
Positive anti-dsDNA Ab, n, %	4, 25%	18, 30%		6, 33.3%	36, 31.03%	
Positive anti-Sm Ab, n, %	4, 25%	7, 11.67%	–	6, 33.33%	22, 18.97%	–
SLEDAI score, median (IQR)	3 (2–6.5)	4 (0–11.75)	–	4 (2–7.75)	8 (2–12)	–
Renal biopsy, n, % a						
Class II		6, 10%			6, 5.17%	
Class III		13, 21.67%			17, 14.66%	
Class IV		20, 33.33%			42, 36.21%	
Class V		9, 15%			21, 18.1%	
Class V + III/IV		12, 20%			30, 25.86%	

Abbreviations: SLE: systemic lupus erythematosus; LN: lupus nephritis; SBP: systolic blood pressure; DBP: diastolic blood pressure; eGFR: glomerular rate filtration; ANAs: antinuclear antibodies; anti-dsDNA: anti-double-stranded DNA; SLEDAI: systemic lupus erythematosus disease activity index. a: lupus nephritis were confirmed by renal biopsy and classified by current International Society of Nephrology/Renal Pathologic Society (ISN/RPS) 2003 criteria.

### 3.2. Plasma sTNF-RI levels were increased in non-LN patients and LN patients

**Figure 1.**
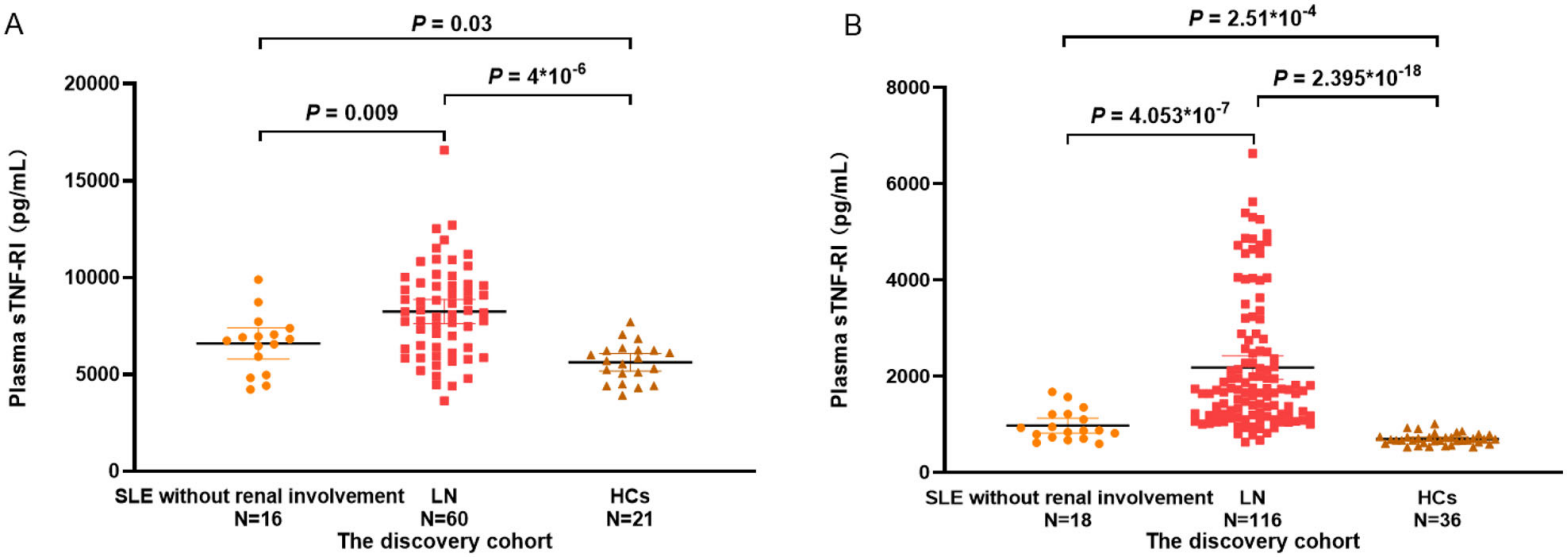
Plasma sTNF-RI levels in SLE without renal impairment patients, LN patients and healthy controls (HCs) of the discovery cohort (A) and in the replication cohort (B).

Of the 21 inflammatory factors tested, plasma sTNF-RI levels differed significantly in the discovery cohort. The plasma sTNF-RI levels were significantly higher in LN patients [8220.94 (6338.67–9645.84) pg/ml] than non-LN patients [6791.14 (5210.08–7308.11) pg/ml] (*p* = .009) and HCs [5706.72 (4779.93–6257.09) pg/ml] (*p* = 4 × 10^−6^). sTNF-RI levels were higher in the non-LN group than in the HCs (*p* = .03) (Figure 1(A)). The finding was confirmed in the independent replication cohort. ELISA determined that the plasma sTNF-RI levels of LN patients [1714.91 (1192.87–2704.57) pg/ml] were significantly higher than non-LN patients [870.58 (721.84–1208.55) pg/ml] (*p* = 4.053 × 10^−7^) and HCs [677.87(615.31–739.1) pg/ml](*p* = 2.395 × 10^−18^) and differed significantly between non-LN patients and HCs (*p* = 2.51 × 10^−4^) (Figure 1(B)).

**Figure 2.**
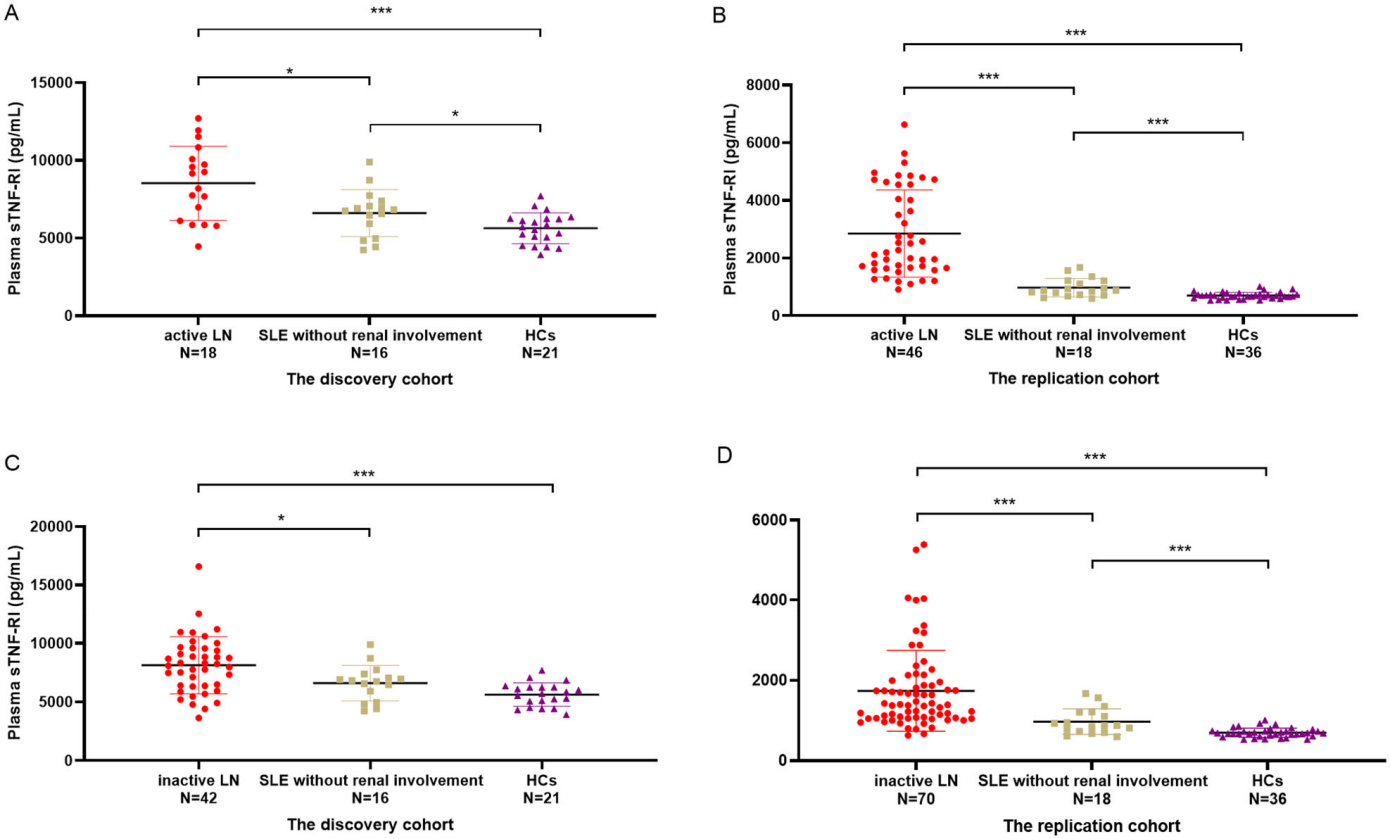
Plasma sTNF-RI levels in active LN patients (SLEDAI > 9), SLE without renal impairment patients and HCs of the discovery cohort (A) and in the replication cohort (C), plasma sTNF-RI levels in inactive LN patients (SLEDAI ≤ 9), SLE without renal impairment patients and HCs of the discovery cohort (B) and in the replication cohort (D). SLEDAI: SLE disease activity, *p < .05, ****p < .001.

Because the disease activity was significantly different in the LN groups, we analyzed the difference between active LN patients (SLEDAI > 9) and inactive LN patients (SLEDAI ≤ 9). In the discovery cohort, we did not observe significant changes between active LN patients and inactive LN patients (*p* = .508). We noticed that the plasma sTNF-RI levels were significantly higher in active LN patients or in inactive LN patients than in HCs or non-LN patients (*p* < .001 and *p* = .021, respectively, in active LN; *p* < .001 and *p* = .017, respectively, in inactive LN). In the replication cohort, the plasma sTNF-RI levels gradually increased in HCs, non-LN patients, inactive LN patients and active LN patients. A significant difference was found between every two groups (*p* < .001) (Figure 2).

The level of plasma sTNF-RI was not different in different types of LN patients. Specifically, the expression of plasma sTNF-RI did not differ between proliferative LN patients (class III/IV, class V + III or IV) and membranous LN (class II or V) (Supplemental Material Table 1, Figures 1 and 2].

### 3.3. Increased plasma sTNF-RI levels were associated with increased disease activity, decreased renal function in SLE patients and the level of urine protein in LN patients

We compared the plasma sTNF-RI levels between SLE patients with and without particular clinical features. In the discovery cohort, plasma sTNF-RI levels in SLE patients with serositis or thrombocytopenia were significantly higher than those in SLE patients without serositis or thrombocytopenia (serositis *p* = .015; thrombocytopenia *p* = .019). The SLE patients without anti-Sm antibodies showed significantly increased plasma sTNF-RI levels compared with those with anti-Sm antibodies (*p* = .039). However, this finding could not be verified in the replication cohort (Table 2).

**Table 2.** Plasma sTNF-RI levels in the presence or absence of manifestations in SLE patients.

Characteristics	The discovery cohort			The replication cohort		
	sTNF-RI *(pg/mL)*			sTNF-RI *(pg/mL)*		
	Presence (n)	Absence (n)	*p*	Presence (n)	Absence (n)	*p*
Malar rash	6873.69 (5347.45–8082.09) (*n* = 8)	7771.65 (6163.15–9574.56) (*n* = 68)	.204	1282.66 (777.17–1657.85) (*n* = 14)	1658.28 (1106.7–2563.51) (*n* = 120)	.002
Arthritis	10,264.76 (6124.06–11,377.32) (*n* = 4)	7707.11 (6163.15–9229.42) (*n* = 72)	.193	1914.9 (995.83–3012.73) (*n* = 6)	1612.51 (1095.5–2338.95) (*n* = 128)	.683
Serositis	9491.71 (7505.03–11,014.25) (*n* = 14)	7438.64 (5942.57–8925.46) (*n* = 62)	**.015**	1290.26 (989.35–3418.53) (*n* = 29)	1653.2 (1111.34–2234.27) (*n* = 105)	.825
Fever	6957.01 (4972.53–8243.75) (*n* = 7)	7768.46 (6216.58–9570.48) (*n* = 69)	.907	1400.38 (1062.34–2645.93) (*n* = 1)	1637.97 (1085.95–2443.39) (*n* = 133)	.88
Leukopenia	6957.01 (4972.53–8243.75) (*n* = 7)	7768.46 (6216.58–9570.48) (*n* = 69)	.232	1745.88 (1353.23–4637.57) (*n* = 15)	1587.05 (1063.31–2272.38) (*n* = 119)	.091
Lyphopenia	8328.85 (6835.21–9628.29) (*n* = 29)	7486.4 (5922.73–9159.02) (*n* = 47)	.229	1637.97 (1043.12–2576.3) (*n* = 49)	1587.05 (1099.37–2415.67) (*n* = 85)	.563
Thrombocytopenia	9556.19 (8679.83–10,602.45) (*n* = 6)	7507.24 (5942.57–9182.49) (*n* = 70)	**.019**	1673.53 (1219.19–4547.11) (*n* = 39)	1637.97 (1082.37–2193.43) (*n* = 95)	.208
Decreased C3	7733.96 (6569.79–9730.53) (*n* = 19)	7768.46 (5935.96–9172.86) (*n* = 57)	.483	1637.97 (1064.52–2859.11) (*n* = 49)	1637.97 (1099.37–2317.67) (*n* = 85)	.794
Decreased C4	7528.08 (6569.79–9730.53) (*n* = 19)	7774.83 (6029.45–9317.36) (*n* = 57)	.938	1516.26 (1006.43–4011.42) (*n* = 39)	1637.97 (1149.45–2275.12) (*n* = 95)	.833
Anti-ANA	7745.04 (6216.58–9570.48) (*n* = 69)	7390.89 (5949.19–9381.84) (*n* = 7)	.907	1637.97 (1100.32–2333.19) (*n* = 126)	1616.68 (919.24–2443.39) (*n* = 8)	.693
Anti-dsDNA Ab	9035.21 (6715.75–10,283.27) (*n* = 22)	7438.64 (5942.57–8847.72) (*n* = 54)	.062	1729.1 (1168–3298.87) (*n* = 42)	1572.21 (1065.69–2254.69) (*n* = 92)	.218
Anti-Sm Ab	6109.72 (4789.18–7774.83) (*n* = 11)	7968.16 (6454.51–9570.48) (*n* = 65)	**.039**	1446.36 (1165.71–3008.59) (*n* = 28)	1637.97 (1062.12–2387.95) (*n* = 106)	.904

Abbreviations: SLE: systemic lupus erythematosus; eGFR: glomerular rate filtration; ANAs: antinuclear antibodies; dsDNA: anti-double-stranded DNA; SLEDAI: systemic lupus erythematosus disease activity index.

Bold values are statistically significant at *p* < .05.

**Figure 3.**
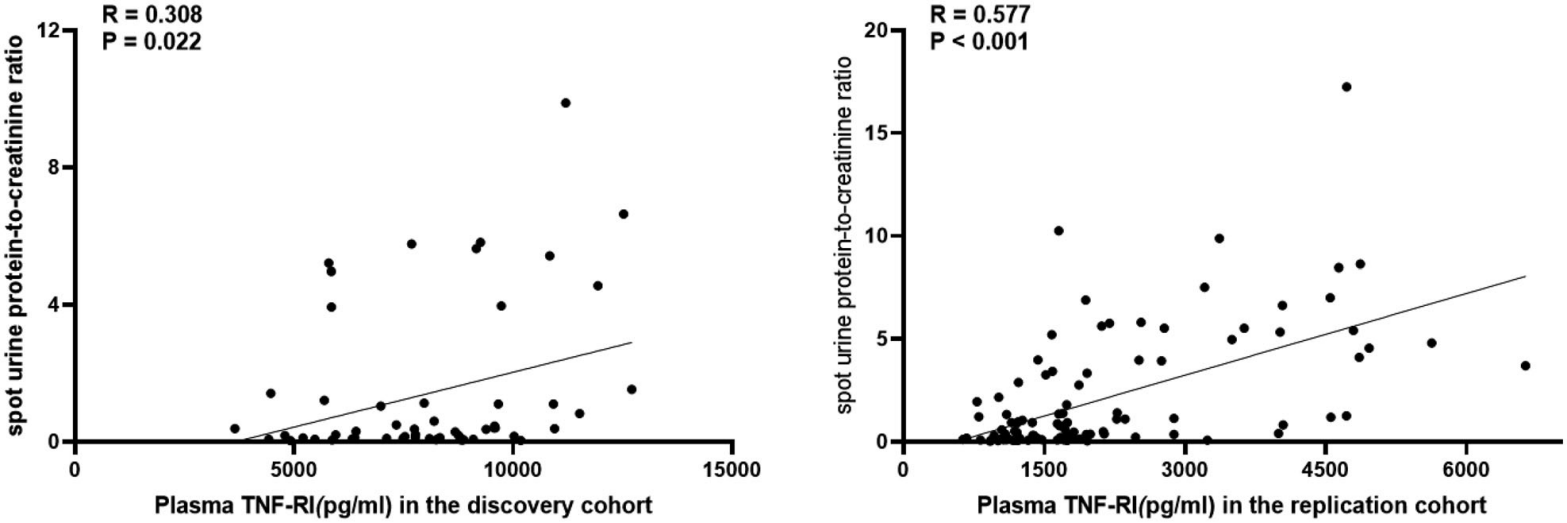
Correlation of plasma sTNF-RI and urine protein-to-creatinine ratio in LN patients of the discovery cohort and in the replication cohort.

**Figure 4.**
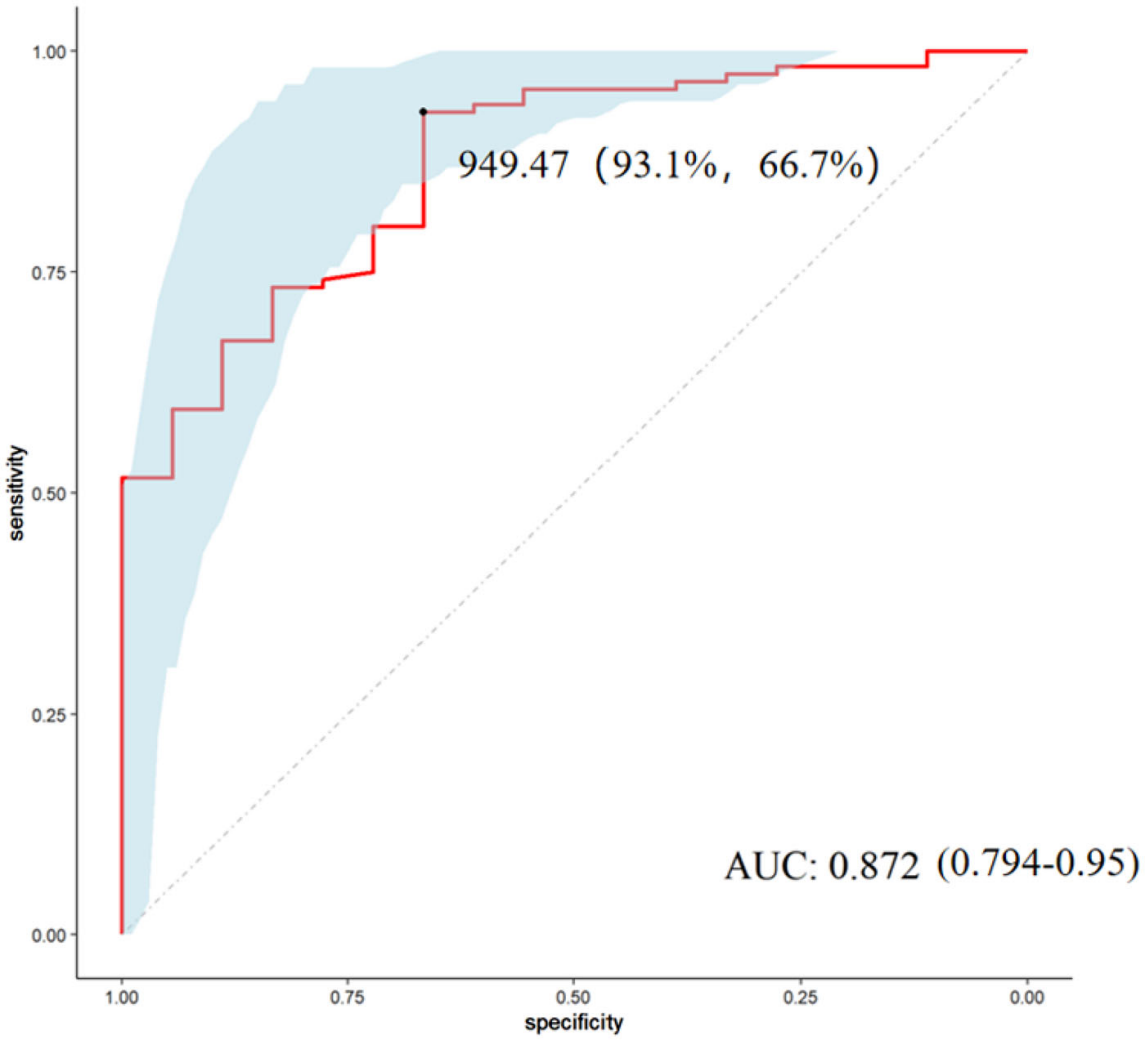
ROC of TNF-RI in differentiating LN patients from SLE without renal impairment patients. ROC: receiver operating characteristic; AUC: the value of the area under the ROC curve.

In the discovery cohort, we found that the level of plasma sTNF-RI was positively correlated with SLEDAI, serum creatinine, urea, uric acid, systolic blood pressure and total cholesterol, while it was inversely correlated with eGFR-EPI, eGFR-MDRD and serum albumin. In the replication cohort, we found that the level of plasma sTNF-RI was positively correlated with SLEDAI, anti-dsDNA Ab, serum creatinine, urea, uric acid, systolic blood pressure, diastolic blood pressure, total cholesterol, triglycerides, and low-density lipoprotein, while it was inversely correlated with C3, eGFR-EPI, eGFR-MDRD and serum albumin. We also recalculated the SLEDAI of LN patients excluding the renal domains, and our data showed that plasma sTNF-RI was positively correlated with SLEDAI (Supplemental Material Table 2, Figures 3 and 4].

To assess the association of plasma sTNF-RI concentrations with renal function decline, we divided LN patients with eGFR-EPI < 90 mL/min/1.73 m^2^ into two groups: chronic acute renal failure (CRF) groups and acute renal failure (ARF) groups. In the replication cohort, plasma sTNF-RI levels were significantly higher in the ARF group than in the CRF group (25 *vs.* 14, 3837.05 (2407.99–4858.57) *vs.* 2136.66 (1513.4–4003.82, *p* = .007).

One patient in the discovery cohort and 1 patient in the replication cohort lacked physical urine examination because of anuria from end-stage renal disease, and 5 patients in the discovery cohort and 10 patients in the replication cohort lacked urine spot urine protein-to-creatinine ratio. Our data showed that in the discovery cohort, LN patients who had urine protein greater than 1+ showed a higher incidence of plasma sTNF-RI levels [17 *vs.* 42, 9578.65 (7329.79–11,021.64) *vs.* 7771.65 (6069.59–8925.46), *p* = .016]. A more detailed analysis demonstrated that the level of plasma sTNF-RI was positively correlated with the spot urine protein-to-creatinine ratio (Spearman’s Rho correlation = 0.308, *p* = .022). This finding was confirmed in our replication cohort. In the replication cohort, LN patients who had urine protein greater than 1+ showed a higher incidence of plasma sTNF-RI levels [43 *vs*. 72, 2747.84 (1653.2–4547.11) *vs.* 1412.44 (1085.55–1861.94), *p* < .001]. The level of plasma sTNF-RI was positively correlated with the urine protein-to-creatinine ratio (Spearman’s Rho correlation = 0.577, *p* < .001) (Figure 3).

### 3.4. Development of a disease diagnosis model for LN patients from non-LNs and active SLE from inactive SLE

To further evaluate the risk factors for LN in SLE patients, we performed univariate logistic regression in the validation cohort, and the model results showed that high TNF-RI, high SBP, low C4 and high UA were risk factors for LN in SLE patients. These factors were included in the multivariate logistic regression model, and the results showed that high TNF-RI was an independent risk factor for LN in SLE patients (Table 3). The ROC results suggested that the use of TNF-RI to distinguish LN from SLE patients had good efficacy [the value of the area under the ROC curve (AUC) 0.872, 95% CI 0.794–0.950, cutoff value 949.47 pg/ml, sensitivity 93.1%, and specificity 66.7%] (Figure 4).

**Table 3.** Logistic regression analysis in differentiating LN patients from no-LNs.

Characteristics	Univariable		Multivariable	
	OR (95%CI)	*p*	OR (95%CI)	*p*
TNF-RI (>949.47 pg/ml)	19.464(5.842–64.849)	**<.001**	10.918(2.711–43.975)	**.001**
SBP (>126.74 mmHg)	12.879(1.658–100.04)	**.015**		
DBP (>80.477 mmHg)	2.265(0.758–6.672)	.143		
Low C3	1.90(0.699–5.166)	.208		
Low C4	2.867(1.041–7.895)	**.042**		
Gender (female)	0.479(0.103–2.229)	.348		
Age (>34.28 year)	1.625(0.571–4.625)	.363		
Uric acid (>312.11 umol/l)	6.968(1.532–31.686)	**.012**		
Positive anti-dsDNA Ab	0.90(0.313–2.587)	.845		
Positive anti-Sm Ab	0.468(0.158–1.384)	.170		
High SLEDAI (>9)	2.30 (0.713–7.424)	.164		

Abbreviations: LN: lupus nephritis; SBP: systolic blood pressure; DBP: diastolic blood pressure.

Bold values are statistically significant at *p* < .05.

**Figure 5.**
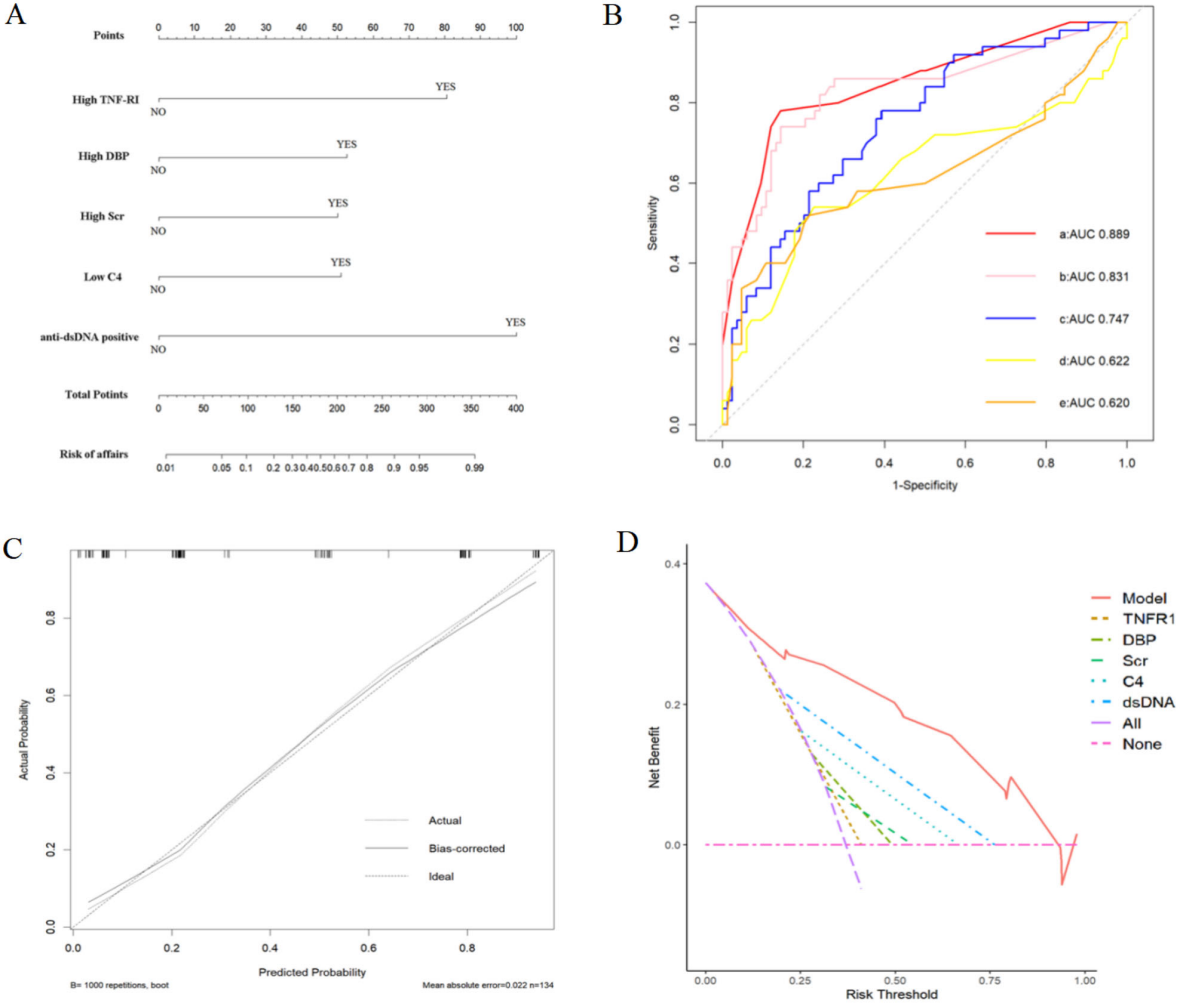
(A) Nomogram based on the active SLE diagnosis model; (B) ROC curves comparing the nomogram (a) with anti-dsDNA (b), TNF-RI (c), serum creatinine (Scr) (d) and diastolic blood pressure (DBP) (e); (C) calibration curve of the nomogram; (D) DCA of the nomogram. ROC: receiver operating characteristic; AUC: the value of the area under the ROC curve; DCA: decision curve analysis.

To identify the risk factors for SLE patients with high disease activity (SLEDAI > 9), we performed univariate logistic regression in the validation cohort. The model results showed that high TNF-RI, high SBP, high DBP, high Scr, low C3, low C4, high UA and anti-dsDNA positivity were risk factors for SLE patients with high disease activity (SLEDAI > 9). In the multivariate logistic regression model, the results showed that high TNF-RI, high DBP, high serum creatinine, low C4 and anti-ds-DNA positivity were independent risk factors for active SLE patients (Table 4). We depicted a nomogram to visualize the model (Figure 5(A)). Next, we plotted ROC curves to assess relative accuracies (Figure 5(B)). The results showed that the ROC curve based on the obtained potential risk factors identified by multivariate logistic regression analysis had the best efficiency, with an AUC of 0.889 (95% CI 0.830–0.948), a specificity of 85.7% and a sensitivity of 78%. The calibration curve indicated that the model had great calibration capability (Figure 5(C)). The results of decision curve analysis (DCA) showed that the established nomogram had the best net benefit and a wide range of threshold probabilities (Figure 5(D)).

**Table 4.** Logistic regression analysis in differentiating active SLE from inactive SLE.

Characteristics	Univariable		Multivariable	
	OR (95%CI)	*p*	OR (95%CI)	*p*
TNF-RI (>949.47 pg/ml)	5.217(1.14–23.872)	**.033**	7.497(1.166–48.224)	**.034**
SBP (>126.74 mmHg)	2.935(1.414–6.092)	**.004**		
DBP (>80.477 mmHg)	2.486(1.213–5.093)	**.013**	2.973(1.036–8.534)	**.043**
Serum creatinine (>126.74 mmol/l)	2.587(1.159–5.777)	**.02**	3.415(1.072–10.883)	**.038**
Low C3	5.982(2.761–12.961)	**<.001**		
Low C4	5.917(2.63–13.311)	**<.001**	5.13(1.531–17.189)	**.008**
Gender （female）	0.941(0.39–2.272)	.893		
Age (>34.28 year)	1.239(0.609–2.521)	.554		
Uric acid (>312.11 umol/l)	2.915(1.413–6.017)	**.004**		
Positive anti-dsDNA Ab	13.156(5.472–31.629)	**<.001**	13.402(4.128–43.508)	**<.001**
Positive anti-Sm Ab	1.944(0.837–4.517)	.122		

Abbreviations: SLE: systemic lupus erythematosus; SBP: systolic blood pressure; DBP: diastolic blood pressure; Anti-dsDNA: anti-double-stranded DNA; SLEDAI: systemic lupus erythematosus disease activity index.

Bold values are statistically significant at *p* < .05.

### 3.5. Renal TNF-RI expression increased in LN patients

**Figure 6.**
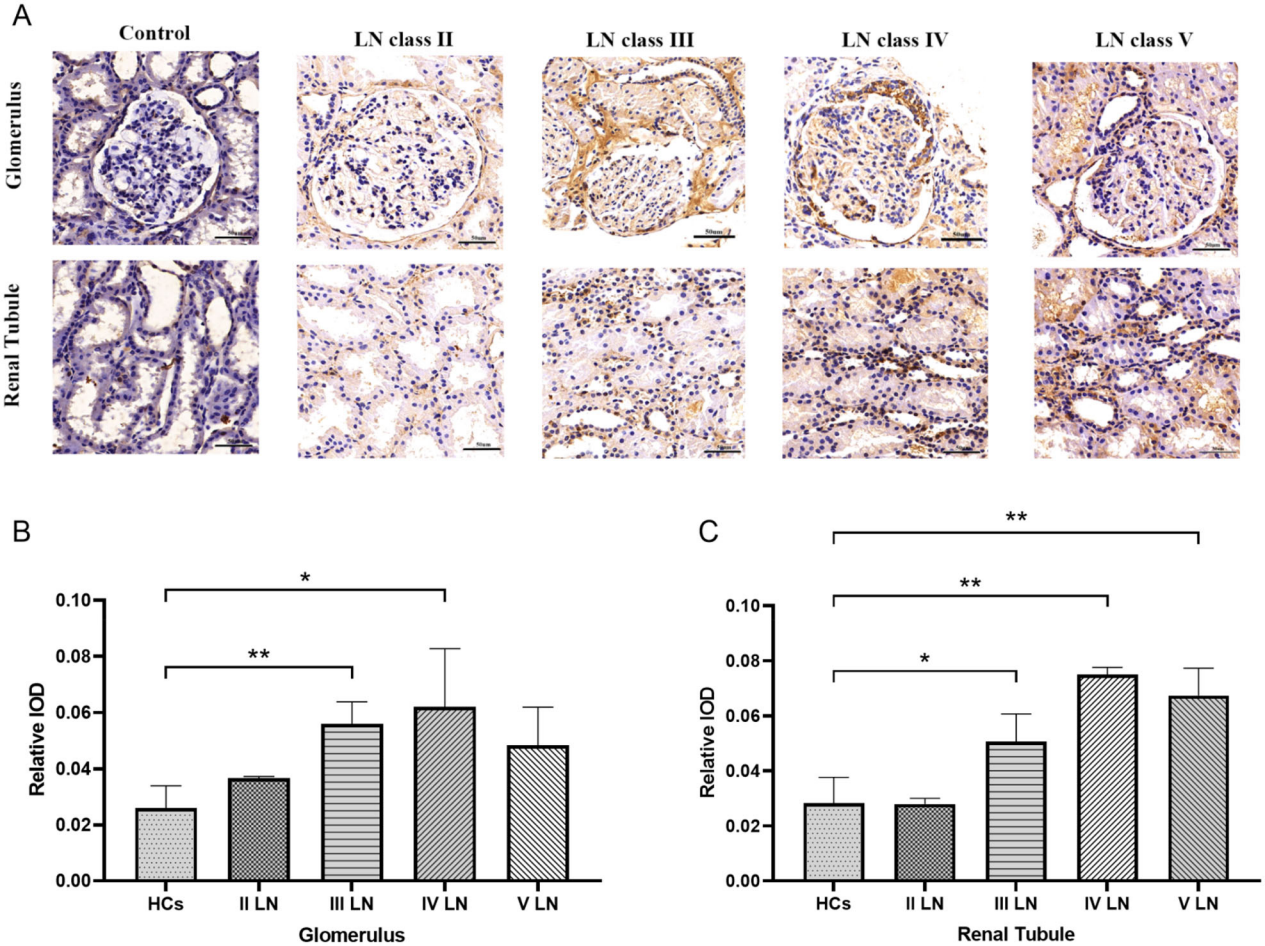
Renal TNF-RI expression in LN patients and controls. (A) Images show TNF-RI immunostaining in the glomeruli and tubulointerstitium. A representative image was shown (scale bar 50 µm); The positive area was quantified with different types and healthy control in glomerulus (B) and renal tubule (C).*p < .05、**p < .01.

IHC results showed that TNF-RI was present predominantly in the nucleus. The expression levels of TMF-RI among different types of LN and the control group are listed in Figure 6. In the normal kidney and type II LN, TNF-RI was faintly stained in the tubulointerstitium and the glomeruli. TNF-RI in the glomeruli of patients with proliferative LN increased significantly. In contrast, the TNF-RI-positive area was significantly higher in the renal tubules of patients with type III, IV and V LN. (Figure 6).

## 4. Discussion

We performed a discovery-replication study to investigate the association between plasma sTNF-RI levels and SLE patients in the Chinese population. Our data showed that plasma sTNF-RI could distinguish between SLE patients with and without renal involvement. Moreover, plasma sTNF-RI was closely related to disease activity and renal function in SLE patients and the level of urine protein in LN patients.

Previous studies reported that TNF-RI could be a new biomarker for early renal decline in patients with type 2 diabetes and that serum sTNF-RI was correlated with plasma creatinine values in patients with sepsis syndrome [16–19], but relatively few investigations have evaluated the function of TNF-RI in patients with LN. As the predominant mediator of TNF signaling, increasing evidence supported the important role of TNF-RI signaling in the pathogenesis of SLE [20]. TNF-RI is expressed in a wide variety of cells, and it is present primarily in glomeruli and peritubular endothelial cells [21]. TNF-RI can promote both proinflammatory and proapoptotic functions, while chronic inflammation is postulated to be involved in deteriorating renal function [22,23]. A large body of evidence suggests that serum sTNF-RI is associated with inflammatory renal disease [24–26]. A previous study reported that serum sTNF-RI levels were associated with renal interstitial fibrosis in patients with glomerulopathies [27]. High levels of TNF-RI in the circulation could activate the production of proinflammatory cytokines and chemokines, which may induce direct renal injury [28]. Previous studies reported higher levels of urinary and serum TNF-RI in LN patients than in HCs and SLE patients. Moreover, TNF-RI was strongly correlated with disease activity in SLE patients and the level of urine protein in LN patients [9,29]. In our study, we set more stringent emission standards for renal impairment. We excluded patients who presented proteinuria during the course of the disease in the SLE without renal impairment group, and we still obtained the same result. Moreover, TNF-RI was associated with renal damage and disease activity after adjusting for potential confounding factors. Our data also showed that plasma sTNF-RI levels were significantly higher in LN patients with ARF than in LN patients with CRF. Furthermore, sTNF-RI could reportedly contribute directly to microvascular kidney injury, which in turn can lead to a sharp decline in renal function. The specific mechanism remains to be further studied. A particularly interesting novel finding was that plasma sTNF-RI levels and urine protein in LN patients were positively correlated. sTNF-RI is excreted in urine, so higher levels of TNF-RI may also indicate glomerular hypofiltration. However, an important consideration was that we also found such differences in the levels of plasma sTNF-RI between SLE patients without renal involvement and HCs, and the urine protein level of the latter was similar to that of HCs. We speculated that plasma TNF-RI might represent renal dysfunction more efficiently and sensitively than urine protein. This finding still merits further evaluation. Moreover, plasma sTNF-RI showed high specificity and sensitivity for the diagnosis of renal involvement and disease activity in SLE patients in our data.

As in previous reports [30], we found that glomerular TNF-RI was strongly increased in proliferative LN. We also observed that TNF-RI expression was increased, especially in renal tubular epithelial cells, in LN patients. Tubular damage plays an important role in the pathophysiology and progression of LN. Proximal renal tubular epithelial cells (PTECs) play an active role in the initiation of immune-mediated injury in LN [31]. However, the role of TNF-RI in the renal tubules has been neglected. Further studies are needed.

Our study also had some limitations. First, it is a monocentric trial with a small sample size. Second, because of the lower sensitivity of ACR criteria, patients with very recent onset of the disease or with less common manifestations may be missed.

As described previously, we suggest that plasma sTNF-RI could be used in clinics as a noninvasive and useful biomarker for SLE disease activity and prediction of renal involvement.

## Supplementary Material

Supplemental Material

## Data Availability

The data that support the findings of this study are available on request from the first author and corresponding author underlying reasonable request.
