# Supplementary material for: Plasma soluble tumor necrosis factor receptor I as a biomarker of lupus nephritis and disease activity in systemic lupus erythematosus patients
Source: Ren Fail. 2023 Mar 22;45(1):2174355. doi: 10.1080/0886022X.2023.2174355 (PMC10035946; doi:10.1080/0886022X.2023.2174355)
Supplement: Supplemental Material [file IRNF_A_2174355_SM8024.pdf]

Supplemental Material Table and Figure

Table 1. The level of plasma sTNF-RI in different types LN.

Characteristics	the discovery cohort sTNF-RI (pg/mL)	the replication cohort sTNF-RI (pg/mL)
Class II	8561.46 (8174.85-9283.74)	1429 (1091-2316)
Class III	9381.84 (7011.53-10505)	1663 (1053-2445)
Class IV	7221.49 (5826.76-9461.48)	1705 (1182-3268)
Class V	8676.92 (6047.5-10096.7)	1587 (1267-1956)
Class V+III/IV	7986.48 (5849-9487.12)	1908 (1233-3734)
Proliferative LN	7775 (6029-9628)	1745 (1186-3198)
Membranous LN	8677 (7768-10020)	1587 (1207-1958)

Information Classification: General

Table 2. Correlation of plasma sTNF-RI and clinical characteristics in SLE patients.

Characteristics	the discovery cohort		the replication cohort	
	Spearman's r	P value	Spearman's r	P value
SBP (mmHg)	0.297	**0.011**	0.373	**< 0.001**
DBP (mmHg)	0.105	0.378	0.236	**0.006**
Urea (mmol/L)	0.566	**< 0.001**	0.574	**< 0.001**
Serum creatinine (umol/L)	0.552	**< 0.001**	0.397	**< 0.001**
Uric acid (umol/L）	0.419	**< 0.001**	0.494	**< 0.001**
eGFR-EPI (ml/min/1.73m^2)	-0.641	**< 0.001**	-0.604	**< 0.001**
eGFR-MDRD (ml/min/1.73m^2)	-0.544	**< 0.001**	-0.535	**< 0.001**
Serum albumin (g/L)	-0.341	**0.003**	-0.573	**< 0.001**
T-CHO (mmol/L)	0.234	**0.045**	0.378	**< 0.001**
TG (mmol/L）	0.059	0.617	0.205	**0.019**
Anti-dsDNA Abs (U/mL)	0.171	0.139	0.272	**0.002**
C3 (g/L)	-0.105	0.368	-0.209	**0.016**
C4 (g/L)	0.128	0.126	-0.074	0.399
SLEDAI	0.322	**0.005**	0.491	**< 0.001**
SLEDAI*	0.089	**0.021**	0168	**< 0.001**

Abbreviations: SLE: systemic lupus erythematosus; SBP: systolic blood pressure; DBP: diastolic blood pressure; eGFR: glomerular rate filtration; T-CHO: total cholesterol; TG: triglyceride; LDL: low-density lipoprotein; HDL: high-density lipoprotein; Anti-dsDNA: anti-double-stranded DNA; SLEDAI: systemic lupus erythematosus disease activity index; *: SLEDAI excluding the renal domainsms

Information Classification: General

Figure 1. The level of plasma sTNF-RI was no difference in different types lupus nephritis.

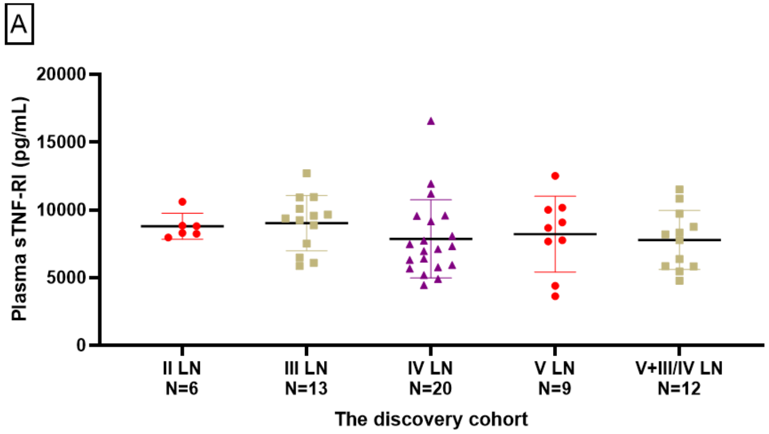

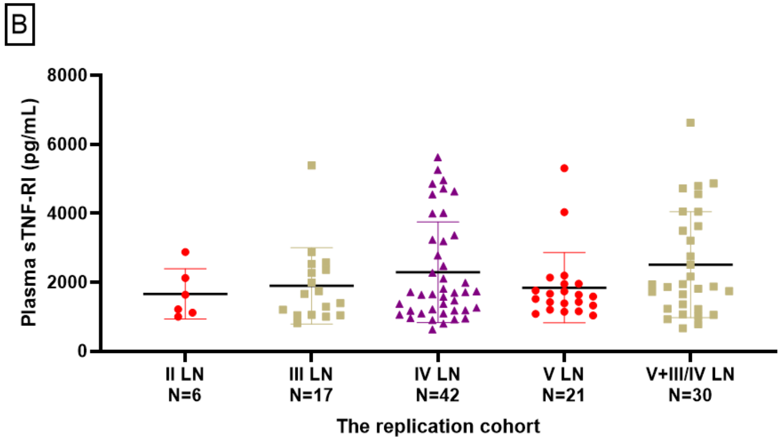

Information Classification: General

Figure 2. The level of plasma sTNF-RI was no difference in proliferative LN (class III/IV, class V+ III or IV) and membranous LN (class II or V).

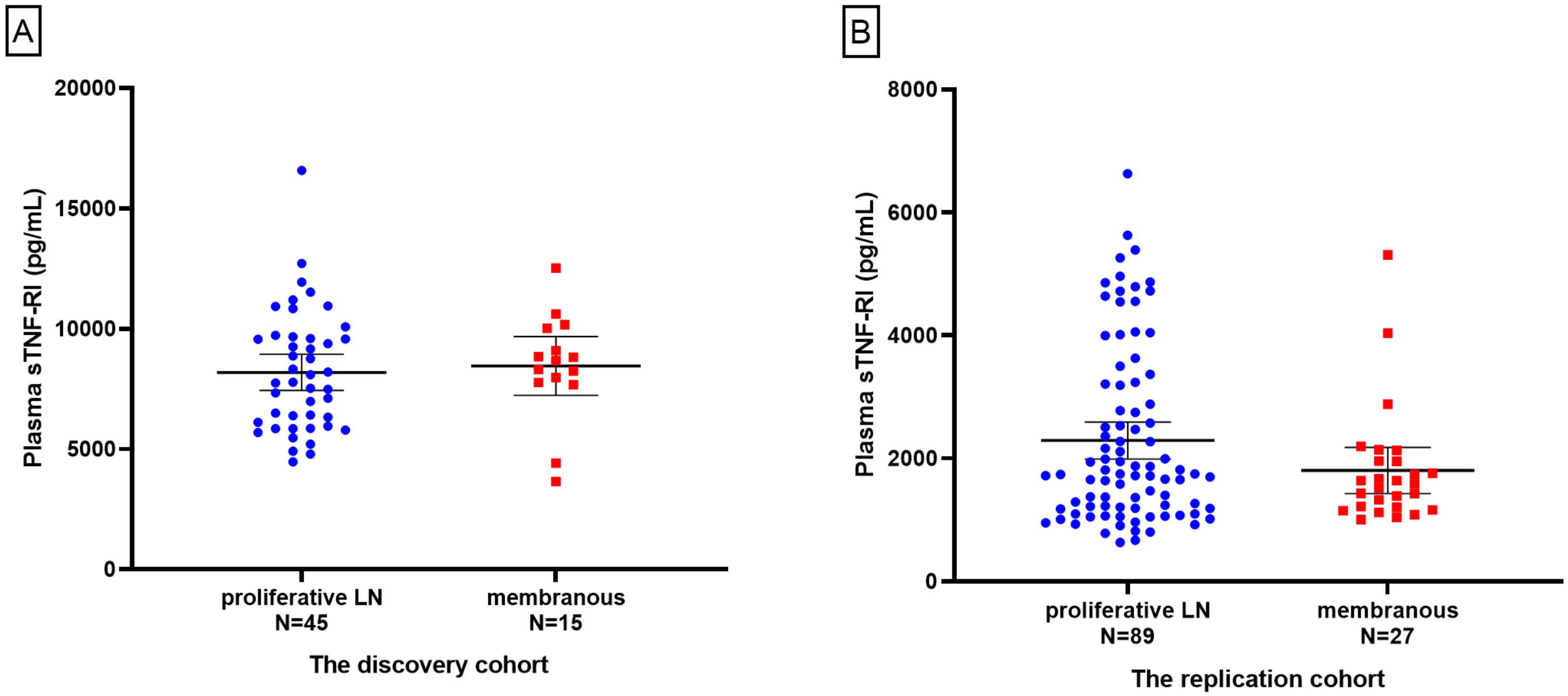

Figure 3. Correlation of plasma sTNF-RI and clinical characteristics in SLE patients of the discovery cohort (A) and in the replication cohort (B).

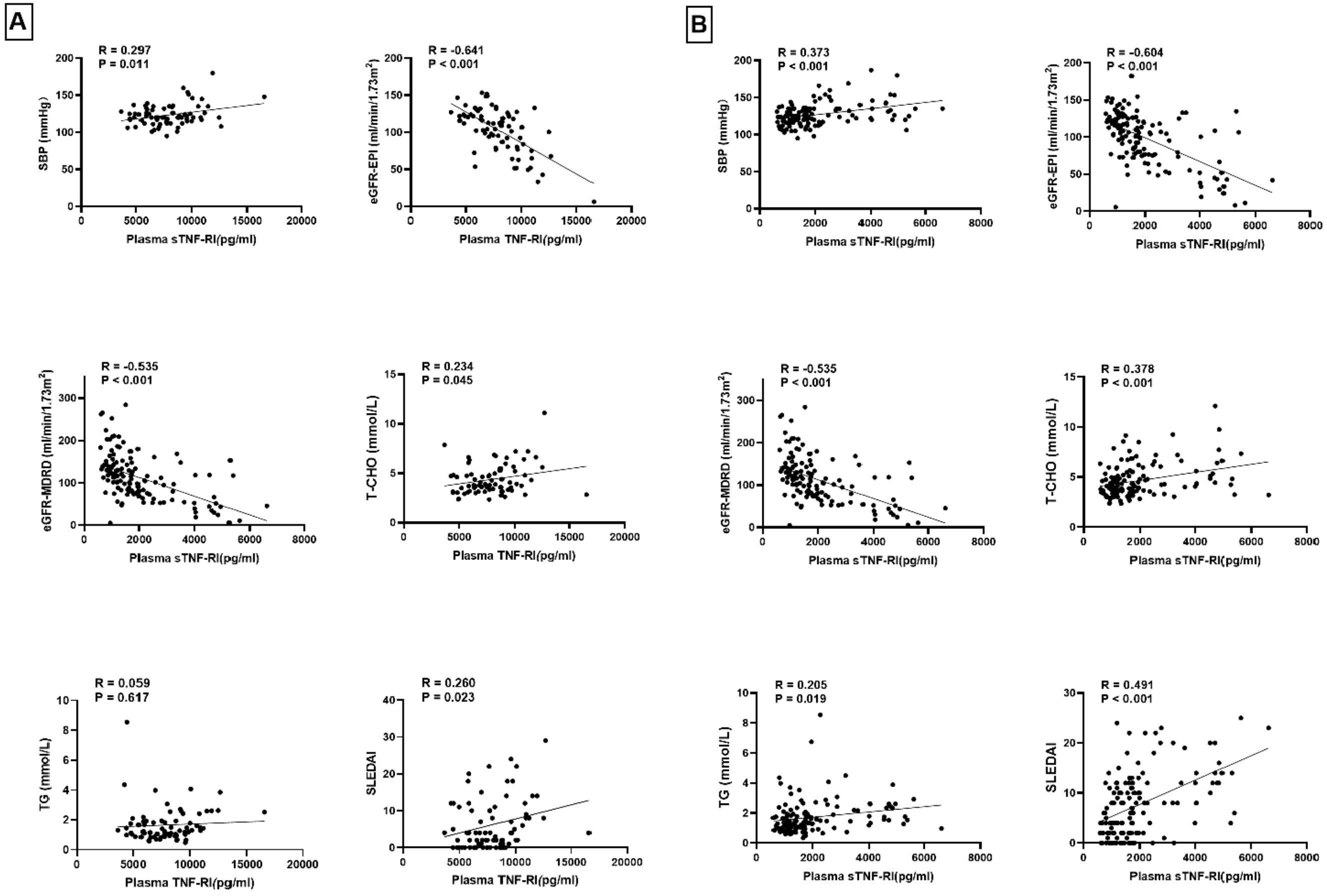

Figure 4. Correlation of plasma sTNF-RI and SLEDAI* (excluding the renal domainsms) in SLE patients of the discovery cohort and in the replication cohort.

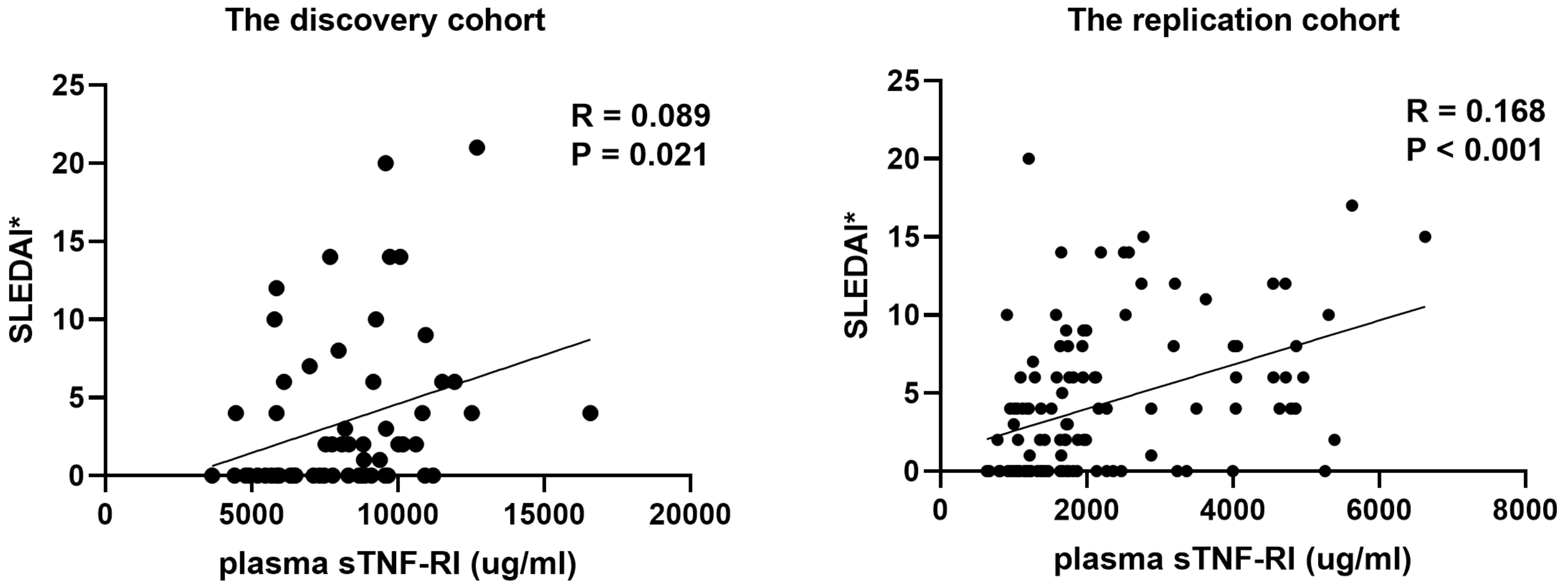

Information Classification: General
